# Establishment and application of a novel isothermal amplification assay for rapid detection of chloroquine resistance (K76T) in *Plasmodium falciparum*

**DOI:** 10.1038/srep41119

**Published:** 2017-01-30

**Authors:** Madhvi Chahar, Neelima Mishra, Anup Anvikar, Rajnikant Dixit, Neena Valecha

**Affiliations:** 1 Division of Epidemiology & Clinical Research, National Institute of Malaria Research, Sector-8 Dwarka, New Delhi, 110077, India

## Abstract

Chloroquine (CQ) resistance in *Plasmodium falciparum* is determined by the mutations in the chloroquine resistance transporter (*Pfcrt*) gene. The point mutation at codon 76 (K76T), which has been observed in more than 91% of *P. falciparum* isolates in India, is the major determinant of CQ resistance. To overcome the limitations and challenges of traditional methods, in this investigation we developed an easy to use loop mediated isothermal amplification (LAMP) protocol for rapid detection of the K76T mutation associated with CQ resistance in *P. falciparum* with naked eye visualization. In- house designed primers were synthesized and optimized to specifically distinguish the CQ resistant mutants of *P. falciparum.* The LAMP reaction was optimal at 61 °C for 60 min and calcein dye was added prior to amplification to enable visual detection. We demonstrate the detection limit of <2 ng/μl respectively, supporting the high sensitivity of this calcein based LAMP method. To the best of our knowledge this is the first report on the establishment of an easy, reliable and cost effective LAMP assay for rapid and specific detection of highly CQ resistance in *P. falciparum* malaria.

*P. falciparum* malaria is still one of the most threatening diseases and fast emergence of resistance to anti-malarial drugs remains one of the challenges to control and eliminate malaria, leading to the renaissance of malaria incidences and death^1^. Currently available molecular tools for rapid detection and tracking of anti-malarial drug resistance are primarily based on the parasite gene markers.

Chloroquine (CQ) resistance, which is mainly caused by the point mutation at codon K76T of the *P. falciparum* chloroquine resistance transporter (*Pfcrt*) gene; is widely used as a molecular marker for the detection of CQ resistance. This molecular marker is highly correlated with increased clinical CQ tolerance and treatment failure^2^,^3^,^4^. However other markers i.e. *P. falciparum* multidrug resistance-1 (*Pfmdr1*) especially codons 86, 184, 1034 and 1042 are known to associated with CQ resistance^5^,^6^.

Although other molecular methods are valuable for anti-malarial drug resistance detection but are, costlier, time-consuming and require highly sophisticated laboratory facilities with trained personnel including DNA sequencing^7^. Since these methods cannot cope with the current demand of field based assays; there is an urgent need to evaluate a new simple, easy to use, rapid, less time consuming and cost effective field based method for detecting anti-malarial drug resistance.

In recent years various isothermal amplification based techniques have been developed with distinct features which are widely used for the detection of various pathogenic species^8^,^9^,^10^. Loop mediated isothermal amplification assay (LAMP) is a fast emerging innovative isothermal amplification tool eliminating the use of highly sophisticated and costly thermo cycler based techniques because there is no requirement of denaturation of DNA template, completing whole reaction in a single step^8^. In comparison to other available isothermal amplification methods, LAMP is easy to use as one step method with high sensitivity and specificity^11^. This method employs strand displacement synthesis primed by specially designed five set of primers, two outer (F3, B3) and two inner (FIP, BIP) primers, identifying six distinct regions on the target DNA^8^. In case of blood parasite detection, LAMP has been found superior to the PCR based techniques, especially due to its stability against inhibitory substance present in blood^12^, easy single step amplification reaction, visual detection and high amplification efficiency^13^.

In recent studies, LAMP assay has also been successfully applied for molecular detection and diagnostics of bacterial^14^,^15^, viral^16^,^17^ fungal^18^,^19^,^20^ and parasitic diseases^21^,^22^ in both animals and plants. Several studies investigate loop mediated isothermal amplification assay (LAMP) for the detection of malaria parasite in humans^23^,^24^,^25^,^26^,^27^,^28^,^29^. However the application of LAMP in the field of anti-malarial drug resistance detection has not been tested so far. In fact early detection of anti-malarial drug resistance in an endemic population is one of the key requirements to plan any pilot surveillance project for control/treatment of malaria, but currently available molecular tools face challenges and limitations of rapid detection in the field setup, requiring optimal laboratory setup with trained man power and cost effectiveness. In this investigation we optimized and tested the ability of LAMP strategy as a novel, reliable and economic tool for rapid CQ resistance detection in field settings.

We developed a field based LAMP method for the detection of CQ resistance in *P. falciparum* caused by K76T point mutation of the *Pfcrt* gene as a molecular marker. Our data provide strong evidence that this new LAMP method could be used for early warning of CQ resistant *P. falciparum* in field conditions. We believe that pre-establishment of a novel isothermal amplification molecular assay for K76T detection could also be useful to develop similar technique valuable for the other important molecular markers like K13-propeller region (especially mutation at codon C580Y, R539T, and Y439H) for large-scale surveillance of artemisinin resistance in *P. falciparum*^30^,^31^.

## Results

### Optimization and specificity of in-house designed LAMP Primers

**Figure 1:**
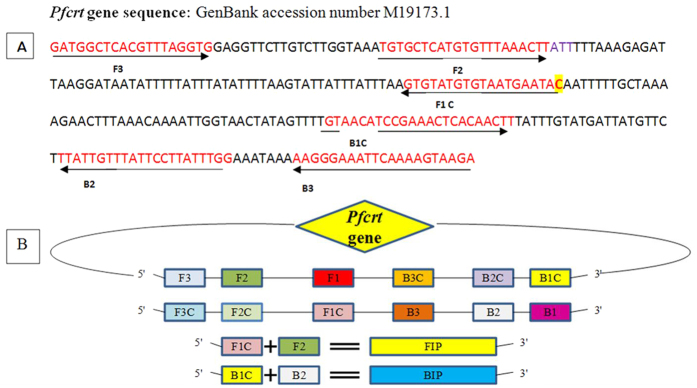
Sequence alignment of *Pfcrt* gene and schematic representation of LAMP primers used in the study. (**A**) Nucleotide sequence alignment of the *Pfcrt* gene target region: Sequences of *Pfcrt* gene primers F3, B3, F1P and B1P used in the LAMP assay. One point mutation indicated by yellow mark (K76T → AAA-ACA) inserted in FIP associated with CQ resistance in *P. falciparum*. Locations of primer-binding regions in the reference sequence (Pf; Gene bank accession no. M19173.1) was indicated by arrows. (**B**) Schematic representation of the *Pfcrt* gene LAMP primers: Construction of the inner primers FIP (F1c + F2) and BIP (B1c + B2) were indicated. F1c and B1c are complementary to F1 and B1.

**Figure 2:**
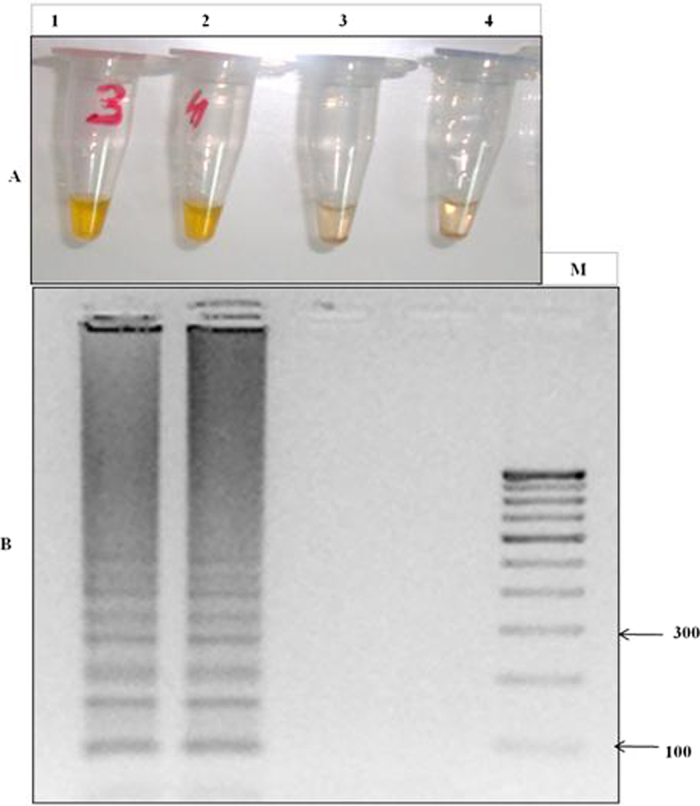
LAMP detection of the K76T mutation through primer set FIPM4 in CQ resistant *P. falciparum* and gel electrophoresis of LAMP products. (**A**) Loop-mediated isothermal amplification (LAMP) detection of the chloroquine-resistant mutants (K76T) of *P. falciparum:* LAMP detection indicated by color change reaction of calcein, bright yellow color was observed when *Pfcrt* gene has K76T mutation but reaction remains light orange if *Pfcrt* gene has no mutation or different mutation. Tube no. 1 indicates positive control strain of *P. falciparum* (RKL9) having K76T mutation and 2 indicate the mutant sample visualized by calcein bright yellow color positive reaction, tube no. 3 indicated wild type sample and tube 4 indicates wild type control strain NF54 used as negative control visualized by calcein light orange color. For the validity of LAMP results mutant control RKL9 and wild type control NF54 was used in each experiment. (**B**) LAMP detection by gel electrophoresis: The positive reaction was indicated by a ladder-like pattern on 3.0% agarose gel; M- 100 bp ladder, Lane 1 and 2 showed ladder like pattern of positive reaction (CQ resistant mutants), while lane 3 and 4 showed no amplification in negative reaction (CQ wild type).

LAMP primers were designed for *Pfcrt* gene encoding K76T point mutation to detect CQ resistance, five primers set (Supplementary Table 1) was designed by using the software Primer explorer V4 program. For the accuracy and specificity of LAMP method the optimization of LAMP primers is essential task. To increase the specificity of LAMP primers for CQ resistance detection, one mismatch was inserted to the forward inner primer (FIP) enabling specific amplification at the target position so the primers would specifically amplify the target position K76T (Fig. 1A,B). Five sets of primers were synthesized and tested for appropriate set of LAMP primers to differentiate the K76T mutant and wild type genomic DNA of *P. falciparum*. Out of five sets only one primer set (FIPM4) qualified for accurate detection of the K76T mutants associated with CQ resistance (Fig. 2). Calcein dye induced color change to bright yellow (positive reaction) in the LAMP in K76T mutant was easy to visual discrimination, when compared to light orange color (negative reaction) in the wild type (Fig. 2A). In post visual discrimination we further reconfirmed the data by gel electrophoresis showing, ladder like pattern in mutant strain (positive control) but missing in wild type (negative control) on 3% agarose gel (Fig. 2B). These results indicated that our in-house designed primer set could be used to distinguish the CQ resistant K76T mutant of *P. falciparum.*

### Optimization of standard conditions and validation of LAMP reaction

**Figure 3:**
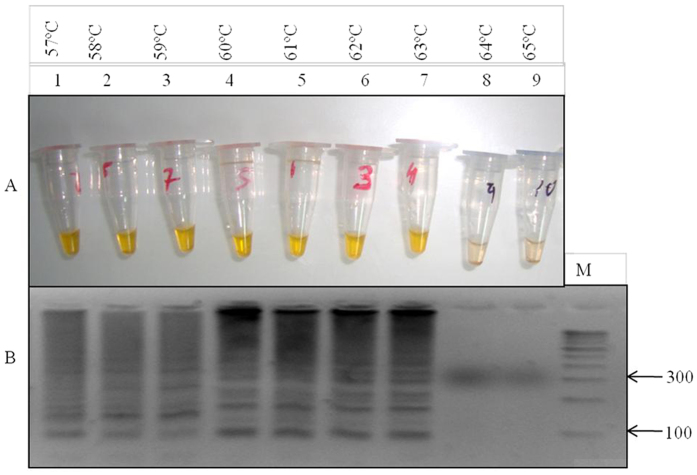
Optimization of LAMP reaction temperature conditions. (**A**) Gel electrophoresis of LAMP product to test the temperature range: M- 100 bp ladder, lane 1 to lane 9 shows the tested temperature range 57–65 °C the best amplification (ladder like pattern) was observed at 61 °C indicated in lane 5. (**B**) Assessment of temperature range by calcein color change visualization: tube no. 1 to 9 indicate the tested temperature range 57–65 °C, there was no color change at 64 °C and 65 °C indicated in tube no. 8 and 9 because the reaction was not amplified at this range and it was reconfirmed on 3% agarose gel as shown in lane 8 and 9.

**Figure 4:**
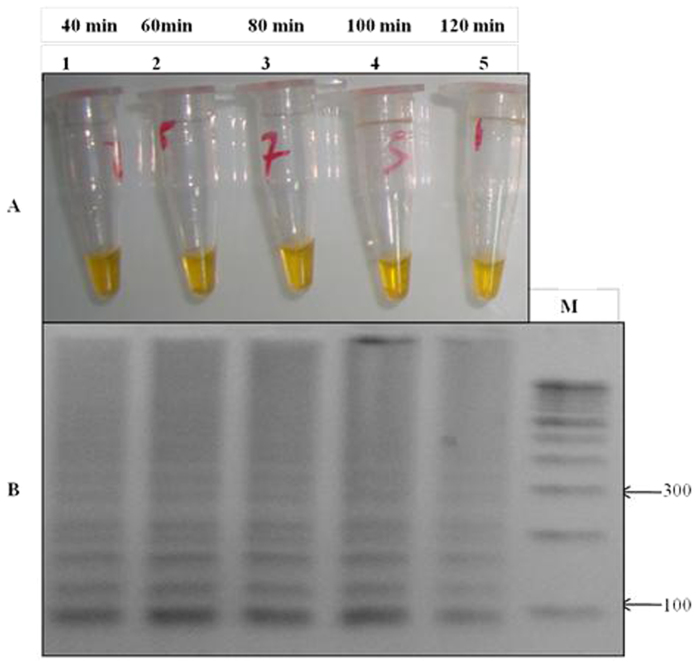
Optimization of LAMP reaction time conditions. (**A**) Assessment was based on calcein color change visualization: Tube no. 1 to 5 shows the tested time range the best color change from light orange to bright yellow was observed at 61 °C min for 60 min, indicated by tube no. 2. (**B**) Gel electrophoresis of LAMP product at different time range: M- 100 bp ladder, lane 1 to 5 showed time range from 40 to 120 min, the best amplification of LAMP product was observed at 60 min indicated in lane 2.

For the optimization of LAMP reaction the initial experiments was performed by using genomic DNA of the mutant control (RKL9), wild type control strain (3D7, NF54, MRC-2) and fifty well characterized samples of *P. falciparum*. To optimize the LAMP assay, a color change in the reaction was monitored in response to different temperature range from 57–65 °C with pre-defined time interval of 40–120 minutes. We observed a highest intensity of color change at 61 °C (Fig. 3A) with an optimal time of 60 min (Fig. 4A) which was successfully corroborated by gel electrophoresis of LAMP product (Figs 3B and 4B), concluding the best optimal LAMP reaction for detection of K76T mutation at 61 °C for 60 min.

### Standardization of LAMP reaction and sensitivity detection

**Figure 5:**
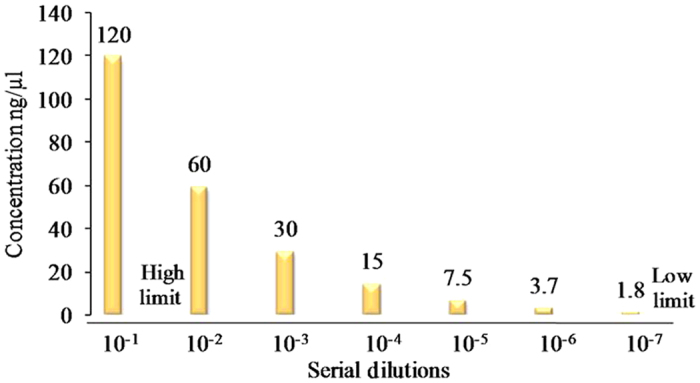
Graphical representation of LAMP detection limit. The lowest limit was detected in 10^−7^ dilution (<2 ng/μl DNA concentration).

**Figure 6:**
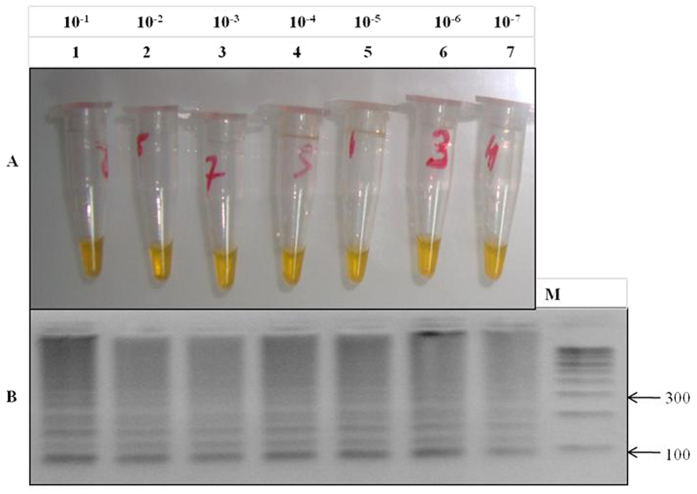
Sensitivity of loop-mediated isothermal amplification (LAMP) versus gel electrophoresis. (**A**) Sensitivity of LAMP for the detection of *Pfcrt* K76T mutation in CQ resistant samples of *P. falciparum*: CQ resistance detection by LAMP and calcein visualization at various dilutions, the minimum limit of LAMP detection is up to 10^−7^ (<2 ng/μl DNA concentration) fold dilutions as shown in tube no. 7. (**B**) Gel electrophoresis of LAMP product to check the sensitivity: M- 100 bp ladder, lane 1 to 7 indicates the dilutions, minimum detection limit showed by light band pattern of lane no. 7 having 10^−7^ dilution (<2 ng/μl DNA concentration).

Following microscopic verification next we evaluated the detection limit of LAMP using serially diluted samples. Our results shows LAMP could detect a limit of 10^−7^ fold dilution, accounting 7 parasites/μl supporting the high sensitivity of LAMP technique. The parasite samples chosen for these dilutions contained initial densities ranging between 0.02 and 1 parasitized cells per 100 red blood cells (referred to as 0.02% and 1% parasitemia, respectively), as determined by microscopic examination. DNA concentration was quantified by using spectrophotometer (Nanodrop Technologies), where a tenfold dilutions were used to perform the reaction. The lowest concentration (<2 ng/μl) of standard DNA detection was successfully observed in 10^−7^ fold dilution (Fig. 5). This confirmation of LAMP sensitivity was monitored by calcein induced color change visualization at different dilutions (Fig. 6A) followed by reconfirmation with 3% gel electrophoresis (Fig. 6B) where showing minimum detection limit visible by band pattern of LAMP amplified product in lane no. 7. Similarly the LAMP reagents were also optimized, allowing best LAMP amplification with 20 μl reaction containing 2.5 U Bst DNA polymerase, 3 mM Mgcl_2,_ 0.8 mM dNTPs, 0.6 M betaine, 2 μM calcein, 1.4 μM FIP and BIP, 0.4 μM F3 and B3 and 2 μl (<2 ng/μl) of DNA. In addition to the calcein color visualization, best ladder like pattern was observed in gel electrophoresis of positive LAMP reaction (DNA having K76T mutants), while no amplification in negative reaction (Fig. 2B) including wild type control (NF54).

### LAMP specificity and accuracy

**Figure 7:**
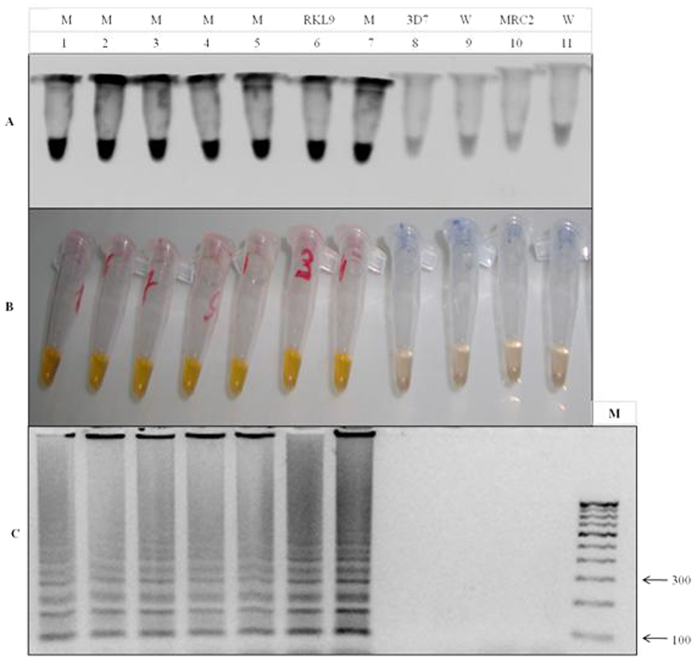
Specificity detection of loop-mediated isothermal amplification (LAMP). (**A**) Assessment was based on calcein color change visualization under Trans UV light: Fluorescence was observed in mutant strains indicated in tube no. 1 to 5 and the reaction was validated by mutant control indicated in tube no. 6. In contrast there was no fluorescence found in the wild type strain indicated by tube no. 9 & 11 and their validation was done by wild type control indicated in tube no. 8 & 10. (**B**) Calcein color change visualization in day light condition: CQ resistant mutant strains of *P. falciparum* is indicated by bright yellow color showed in tube no. 1 to 5 with positive control RKL9 indicated in tube no. 6, in contrast the CQ wild type strain showed light orange color reaction indicated by tube no. 9 and 11, with wild type control 3D7 and MRC2 (tube no. 8 & 10). (**C**) Assessment was based on gel electrophoresis: M- 100 bp ladder, lane 1 to 7 showed amplification in CQ resistant mutants with positive control RKL9 (lane 6) in contrast there was no amplification in wild types indicated in lane 8 to 11 including the wild type control 3D7 and MRC2.

The above demonstration of the successful LAMP assay optimization clearly differentiated the mutant (RKL9) and wild type (3D7, NF54, MRC2) control strains. To better evaluate the efficiency and specificity we further assessed the calcein dye fluorescence in the presence of UV light (Fig. 7A and B). Our results showed dark black coloration appearance in the positive LAMP amplification DNA samples having K76T mutant, reconfirmed by ladder like pattern on gel electrophoresis, but no amplification in the negative reaction (Fig. 7C). A comparative assessment of each LAMP experiments with the inclusion of negative, positive (Pre-validated strains) control samples not only showed high specificity of our test, but also confirmed the 100% accuracy, as validated by sequencing analysis (given below).

### LAMP validation, stability and repeatability test

**Figure 8:**
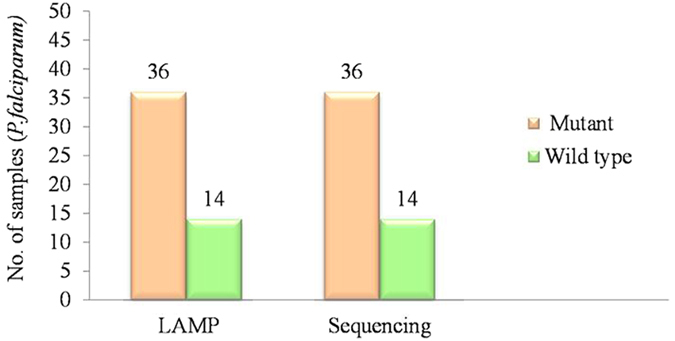
Graphical representation of detected CQ resistant and wild type strains by LAMP and their validation by Sequencing. The figure shows the validation of LAMP analysis by gold standard sequencing method.

Finally, to validate the result of positive LAMP reaction, the final amplification products including mutant control strain RKL9 were subjected to gold standard method of DNA sequencing. Our results showed 100% identity to DNA sequences when compared to the standard mutant and wild type strains (Fig. 8), that optimized LAMP to specifically amplify the *Pfcrt* gene region having K76T mutation. To test the stability, robustness and repeatability of the LAMP technique, 36 known K76T mutants and 14 known wild type samples (with the wild type and mutant controls) from Tripura region (Table S2 and Fig. 8) were tested three times independently by LAMP. All the mutant strains of *P. falciparum* were found positive including the mutant control RKL9 and wild type strains were negative including the wild type control. Analysis was based on calcein- visualization or gel electrophoresis (Figs 2 and 7). Sensitivity, specificity and accuracy of LAMP were compared and statistically analysed with PCR-RFLP and gold standard sequencing results (Table 1). The highest agreement with sequencing gold standard was achieved with the LAMP visual interpretation and LAMP-gel (100%, kappa (κ) = 1.0), showed a similar level of agreement with sequencing (Table 1). The visual interpretation by LAMP seemed to be high with kappa value 1.0 respectively. The κ statistics (range 1, to −1) measures the observed percentage of agreement between tests against what might be expected by chance, ranging from <0 = poor agreement to 1 = perfect agreement. On the basis of results it suggested that establishment of LAMP technique had good stability and repeatability to detect the CQ resistant and wild type samples of *P. falciparum.*

### Application of LAMP for monitoring chloroquine resistant *P. falciparum* as a field based method

The aim of this study was to demonstrate the application of LAMP for rapid monitoring of chloroquine resistant *P. falciparum* in the field conditions. Our technique is based on simple one step amplification procedure, where; results could be analyzed by naked eye within 60 min without using thermal cycler and other standard lab practices. To further verify the application of LAMP, we analyzed a total of 50 well characterized *P. falciparum* samples including 36 mutants (K76T) and 14 wild type strains. In each experiment we included, the mutant control (RKL9) and the wild type control 3D7, NF-54, MRC2 to check the accuracy of LAMP results, cross checked by DNA sequencing (Table S2). The results detected by LAMP were in full agreement to the result of sequencing gold standard method (Table S2 and Fig. 8); verifying that current optimized LAMP strategy is feasible to detect the CQ resistant K76T mutants with limited amount of DNA samples and can be used for rapid monitoring of CQ resistant population of *P. falciparum* in field.

## Discussion

In recent years LAMP strategy has been recognized as a unique and valuable molecular tool enabling fast, simple, accurate and cost effective diagnosis and detection of various bacterial, viral, fungal and parasitic diseases^11^,^14^,^15^,^16^,^17^,^18^,^19^,^20^,^21^,^22^,^23^,^24^,^26^,^32^,^33^. However the application of LAMP strategy to detect anti-malarial drug resistance in *P. falciparum* remains un-reported. To test and validate its suitability of malaria resistance evaluation, we developed a field based simple, easy to use and rapid LAMP assay, enabling highly chloroquine resistant *P. falciparum* detection. For a successful LAMP method, it is always challenging to design and identify highly specific primers, quantifying various conditions and possibilities of one step target DNA amplification. Initially, to identify K76T mutation in *Pfcrt* gene responsible for CQ resistance in *P. falciparum,* we designed and tested a set of five primers; while only one primer set (FIPM4) out of five was found to be most appropriate for the detection of K76T mutation in CQ resistant *P. falciparum* (Table S1 and Fig. 2). Selected loop primers not only accelerated the LAMP reaction by shortening the reaction time in comparison to the original method^34^; but also distinguished the mutant from the wild types, simply by one mismatch addition to the forward inner primer (FIP).

On the basis of selected most appropriate LAMP primer set, the reaction conditions and the concentration of reagents were optimized for successful LAMP assay establishment. In fact, the principle of LAMP results visualization is mainly based on the production of pyrophosphate ions, which binds to metal indicator and form a white precipitate. Therefore the naked eye visualization of LAMP results is possible by adding metal ion as indicator or fluorescent dye i.e. calcein, hydroxynaphthol blue (HNB), SYBR green, calcium chloride, magnesium pyrophosphate and CuSO_4_ etc^35^. Though most of fluorescent intercalating dyes (SYBR green, Picogreen, EvaGreen etc.) can be added after the completion of LAMP reaction, but re-opening of reaction tubes and adding dye may have greater chance of contamination and false results. To avoid the chances of contamination, in this study we added calcein dye in the reaction mix prior to incubation, therefore the reaction was completed in a closed system and the color change detection system doesn’t require any costly equipment. For a successful LAMP reaction simple naked eye visualization of color change from light orange to bright yellow for positive samples, was further confirmed through gel electrophoresis.

As compared to traditional PCR based methods, the developed LAMP is not only easy to use, faster, without requiring any costly instruments and sophisticated lab settings, but the results are also easier to assess with naked eye. Furthermore, in our protocol we observed that a successful LAMP reaction needing an optimal temperature 61 °C incubation, and could be finished within 60 min, for the detection of K76T mutation in *P. falciparum.* Because LAMP is conducted isothermally, requiring heat block of constant temperature and thus it should be useful even for those laboratory conditions unfamiliar with PCR based methods or other molecular analysis. Our independent validation by DNA sequencing of each LAMP experimental data provide strong evidence of robustness, repeatability, specificity of our in-house optimized LAMP protocol with high accuracy and sensitivity, requiring very limited amount i.e. <2 ng/μl of genomic DNA.

Abdul Ghani^36^ suggested the application and possibilities of LAMP as POCTs for the diagnosis of malaria where; several studies followed to evaluate the LAMP assay in the field of malaria diagnosis^23^,^24^,^25^,^26^,^33^. However evaluation of antimalarial drug resistance using LAMP remains un-tested, though it has been well tested to detect resistance in bacteria^37^,^38^,^39^. Towards this goal, our newly developed LAMP was found highly sensitive, specific and efficient as compare to other PCR based molecular methods for rapid and easy detection of CQ resistance in *P. falciparum*.

In conclusion a field based LAMP assay with calcein visualization was established and demonstrated to be simple, easy to use, more sensitive, specific, and found to be very feasible to detect the K76Y mutation in CQ resistant samples of *P. falciparum* than previously used PCR based methods. Therefore the application of this method can be potentially useful for monitoring and early warning of CQ resistance in *P. falciparum* malaria, it could be an effective approach to facilitate anti-malarial resistance detection and tracking in resource-limited countries afflicted with the disease, so that correct and timely treatment can be given to reduce morbidity and mortality associated with the disease. There is need to field test the assay on a larger sample size to assess suitability in field conditions. Moreover we also believe that current optimized technique could be very helpful for rapid optimization of a protocol to detect other drug resistance markers including K13 for artemisinin drug resistance in *P. falciparum*.

## Methods

### *P. falciparum* samples

This study was approved by institutional ethical committee; fifty microscopically examined *P. falciparum* samples were used in this study. The samples were collected from Tripura region, India and chloroquine (CQ) resistant (K76T) mutant control strain (RKL9) and wild type control strain (3D7, NF54 and MRC2) were collected from Malaria Parasite Bank of National Institute of Malaria Research (NIMR), New Delhi, India. These samples were previously characterized by PCR-RFLP for *Pfcrt* K76T mutation associated with CQ resistance in *P. falciparum.*

### Genomic DNA extraction and Quantification

Genomic DNA was extracted from *P. falciparum* samples and control strains by using the QIAamp DNA Blood Mini/Maxi Kit (Qiagen) as per manufacturer’s protocol. In order to quantify the minimum DNA concentration (ng/μl), optical density (OD) of extracted DNA samples were checked by using spectrophotometer (Nanodrop Technologies). A tenfold serial dilutions (10^−1^ to 10^−7^) were used to determine the minimum detected DNA concentration. The LAMP sensitivity was determined up to 10^−7^ fold dilution (<2 ng/μl DNA).

### In-house design LAMP primers

Five sets of primers for LAMP assay were designed by using Primer explorer V4 software (http://primerexplorer.jp/e/). The sequence of *P. falciparum* (GenBank accession number M19173.1) were used to designed the target, we have designed the parameters for most widely reported mutation (K76T) in *Pfcrt* gene associated with molecular detection of CQ resistance. LAMP primers sets and their sequence alignment used in this study are given in Table S1 and Fig. 1.

### Specificity of LAMP primers

To improve the specificity of the in-house designed LAMP primers, one base-pair mismatche was introduced within three nucleotides of the 3’end of the forward inner primer (FIP) (Fig. 1). Thus five sets of LAMP primers were synthesized and tested to distinguish the *Pfcrt* K76T mutants from the wild type. The genomic DNA of mutant control strain RKL9 and wild type control strain 3D7, NF-54, MRC2 were tested to validate the accuracy of LAMP primers. The LAMP was performed at the given standard optimum conditions; each LAMP product was analyzed by gel electrophoresis on 3.0% agarose gel. In addition, the LAMP amplification product visualized by naked eye with the calcein color change reaction from light orange to bright yellow if the required product was present in contrast it remain light orange in the absence of product.

### Optimization of standard LAMP reaction and conditions

The standard LAMP reaction was optimized in a total volume of 20 μl. For the standardization of LAMP reagents, various concentration range were tested for calcein (0.5–4.0 μM), Bst DNA polymerase (1–3 U), dNTPs (0.2–1.5 mM), primers FIP, BIP (0.6–2.0 μM) and F3, B3 (0.2–0.8 μM), betaine (0.3–1 M) and Mgcl_2_ (0.5–4.0 mM) were evaluated. The best LAMP amplification was obtained with 20 μl reaction which contain 2.5 U Bst DNA polymerase, 3 mM Mgcl_2_, 0.8 mM dNTPs, 0.6 M betaine, 2 μM calcein, 1.4 μM FIP and BIP, 0.4 μM F3 and B3 and 2 μl (<2 ng/μl) of DNA. The reaction were incubated at 57 °C, 58 °C, 59 °C, 60 °C, 61 °C, 62 °C, 63 °C, 64 °C and 65 °C to determine the optimal reaction temperature and the LAMP was also performed at different time intervals of 40, 60, 80, 100 and 120 min to determine the optimal reaction time. The standard LAMP assay conditions were optimized at 61 °C for 60 min and then at 90 °C for 7 min to terminate the reaction. The LAMP assay was based on the assessment of calcein color change reaction and pattern of band in gel electrophoresis. To confirm the stability each experiment was repeated thrice having three independent replicates.

### Confirmation of the LAMP results by Sequencing

For the validation and accuracy of LAMP results, CQ resistant samples having *Pfcrt* K76T mutation and all the wild types were confirmed through commercial available sequence service.

### Statistical analysis

All statistical analysis was performed using Statistical Package for the Social Sciences (SPSS) software version 10. Sensitivity, specificity and accuracy of LAMP and PCR-RFLP were comparable with results of sequencing gold standard reference test. In this study we evaluated the LAMP assay using two different methods (sequencing and PCR-RFLP) and each experiment was repeated thrice, leading to three practical modalities for assessing a positive test result.

## Additional Information

**How to cite this article**: Chahar, M. *et al*. Establishment and application of a novel isothermal amplification assay for rapid detection of chloroquine resistance (K76T) in *Plasmodium falciparum. Sci. Rep.* **7**, 41119; doi: 10.1038/srep41119 (2017).

**Publisher's note:** Springer Nature remains neutral with regard to jurisdictional claims in published maps and institutional affiliations.

## Supplementary Material

Supplementary Information

## Figures and Tables

**Table 1:** Senisitivity, specificity and accuracy of LAMP and PCR-RFLP versus gold standard sequencing.

	Measure of agreement with gold standard sequencing (κ)	Sensitivity	Specificity	Accuracy
LAMP- gel	1.00	100%	100%	100%
LAMP- visible	1.00	100%	100%	100%
PCR-RFLP	0.92	97%	94%	96%

Kappa coefficient (κ) statistics range (1 to −1), *p* < 0.0005 measures the observed percentage of agreement between tests. LAMP-gel, Loop mediated isothermal amplification by gel electrophoresis; LAMP-visible, Loop mediated isothermal amplification visualized by naked eye; RFLP, Restriction fragment length polymorphism.

## References

[b1] Murray C. J. . Global malaria mortality between 1980 and 2010: a systematic analysis. Lancet 379, 413–431 (2012). 22305225 10.1016/S0140-6736(12)60034-8

[b2] Ecker A., Lehane A. M., Clain J. & Fidock D. A. PfCRT and its role in antimalarial drug resistance. Trends Parasitol 28, 504–514 (2012). 23020971 10.1016/j.pt.2012.08.002 PMC3478492

[b3] Lakshmanan V. . A critical role for PfCRT K76T in Plasmodium falciparum verapamil-reversible chloroquine resistance. EMBO J 24, 2294–2305 (2005). 15944738 10.1038/sj.emboj.7600681 PMC1173140

[b4] Wellems T. E. & Plowe C. V. Chloroquine-resistant malaria. J Infect Dis 184, 770–776 (2001). 11517439 10.1086/322858

[b5] Folarin O. A. . Chloroquine Resistant Plasmodium falciparum in Nigeria: Relationship between pfcrt and pfmdr1 Polymorphisms, *In-Vitro* Resistance and Treatment Outcome. Open Trop Med J 1, 74–82 (2008). 19953193 10.2174/1874315300801010074 PMC2785046

[b6] Foote S. J. . Several alleles of the multidrug-resistance gene are closely linked to chloroquine resistance in Plasmodium falciparum. Nature 345, 255–258 (1990). 2185424 10.1038/345255a0

[b7] Petersen I., Eastman R. & Lanzer M. Drug-resistant malaria: molecular mechanisms and implications for public health. FEBS Lett 585, 1551–1562 (2011). 21530510 10.1016/j.febslet.2011.04.042

[b8] Notomi T. . Loop-mediated isothermal amplification of DNA. Nucleic Acids Res 28, E63 (2000). 10871386 10.1093/nar/28.12.e63 PMC102748

[b9] Versalovic J. & Lupski J. R. Molecular detection and genotyping of pathogens: more accurate and rapid answers. Trends Microbiol 10, S15–21 (2002). 12377563 10.1016/s0966-842x(02)02438-1

[b10] Vincent M., Xu Y. & Kong H. Helicase-dependent isothermal DNA amplification. EMBO Rep 5, 795–800 (2004). 15247927 10.1038/sj.embor.7400200 PMC1249482

[b11] Mori Y. & Notomi T. Loop-mediated isothermal amplification (LAMP): a rapid, accurate, and cost-effective diagnostic method for infectious diseases. J Infect Chemother 15, 62–69 (2009). 19396514 10.1007/s10156-009-0669-9 PMC7087713

[b12] Thekisoe O. M. . Loop-mediated isothermal amplification (LAMP) assays for detection of Theileria parva infections targeting the PIM and p150 genes. Int J Parasitol 40, 55–61 (2010). 19654009 10.1016/j.ijpara.2009.07.004

[b13] Parida M., Sannarangaiah S., Dash P. K., Rao P. V. & Morita K. Loop mediated isothermal amplification (LAMP): a new generation of innovative gene amplification technique; perspectives in clinical diagnosis of infectious diseases. Rev Med Virol 18, 407–421 (2008). 18716992 10.1002/rmv.593 PMC7169140

[b14] Lee D. . Clinical evaluation of a loop-mediated isothermal amplification (LAMP) assay for rapid detection of Neisseria meningitidis in cerebrospinal fluid. PLoS One 10, e0122922 (2015). 25853422 10.1371/journal.pone.0122922 PMC4390149

[b15] Jun-hai N. . Development and evaluation of a loop-mediated isothermal amplification assay for rapid detection of bacterial blight pathogen (Xanthomonas axonopodis pv. dieffenbachiae) in anthurium. European Journal of Plant Pathology 142, 801–813 (2015).

[b16] Okuda M., Okuda S. & Iwai H. Detection of Cucurbit chlorotic yellows virus from Bemisia tabaci captured on sticky traps using reverse transcription loop-mediated isothermal amplification (RT-LAMP) and simple template preparation. J Virol Methods 221, 9–14 (2015). 25912723 10.1016/j.jviromet.2015.04.014

[b17] Wang X. . Rapid detection of active human cytomegalovirus infection in pregnancy using loop-mediated isothermal amplification. Mol Med Rep 12, 2269–2274 (2015). 25847382 10.3892/mmr.2015.3572

[b18] Duan Y. B. . Development and application of loop-mediated isothermal amplification for detecting the highly benzimidazole-resistant isolates in Sclerotinia sclerotiorum. Sci Rep 5, 17278 (2015). 26606972 10.1038/srep17278 PMC4660316

[b19] Dai T. T. . Development of a loop-mediated isothermal amplification assay for detection of Phytophthora sojae. FEMS Microbiol Lett 334, 27–34 (2012). 22697582 10.1111/j.1574-6968.2012.02619.x

[b20] Niessen L. & Vogel R. F. Detection of Fusarium graminearum DNA using a loop-mediated isothermal amplification (LAMP) assay. Int J Food Microbiol 140, 183–191 (2010). 20442002 10.1016/j.ijfoodmicro.2010.03.036

[b21] Kogovsek P. . LAMP assay and rapid sample preparation method for on-site detection of flavescence doree phytoplasma in grapevine. Plant Pathol 64, 286–296 (2015). 26146413 10.1111/ppa.12266 PMC4480326

[b22] Zhuo X. . Development and application of loop-mediated isothermal amplification assays based on ITS-1 for rapid detection of Toxoplasma gondii in pork. Vet Parasitol 208, 246–249 (2015). 25624074 10.1016/j.vetpar.2015.01.008

[b23] Sattabongkot J., Tsuboi T., Han E. T., Bantuchai S. & Buates S. Loop-mediated isothermal amplification assay for rapid diagnosis of malaria infections in an area of endemicity in Thailand. J Clin Microbiol 52, 1471–1477 (2014). 24574279 10.1128/JCM.03313-13 PMC3993686

[b24] Mohan A. An innovative test for rapid diagnosis of malaria. Natl Med J India 27, 297 (2014). 26037447

[b25] Patel J. C. . Field evaluation of a real-time fluorescence loop-mediated isothermal amplification assay, RealAmp, for the diagnosis of malaria in Thailand and India. J Infect Dis 210, 1180–1187 (2014). 24795480 10.1093/infdis/jiu252 PMC6373533

[b26] Polley S. D. . Mitochondrial DNA targets increase sensitivity of malaria detection using loop-mediated isothermal amplification. J Clin Microbiol 48, 2866–2871 (2010). 20554824 10.1128/JCM.00355-10 PMC2916605

[b27] Yamamura M., Makimura K. & Ota Y. Evaluation of a new rapid molecular diagnostic system for Plasmodium falciparum combined with DNA filter paper, loop-mediated isothermal amplification, and melting curve analysis. Jpn J Infect Dis 62, 20–25 (2009). 19168954

[b28] Han E. T. . Detection of four Plasmodium species by genus- and species-specific loop-mediated isothermal amplification for clinical diagnosis. J Clin Microbiol 45, 2521–2528 (2007). 17567794 10.1128/JCM.02117-06 PMC1951264

[b29] Poon L. L. . Sensitive and inexpensive molecular test for falciparum malaria: detecting Plasmodium falciparum DNA directly from heat-treated blood by loop-mediated isothermal amplification. Clin Chem 52, 303–306 (2006). 16339303 10.1373/clinchem.2005.057901

[b30] Miotto O. . Multiple populations of artemisinin-resistant Plasmodium falciparum in Cambodia. Nat Genet 45, 648–655 (2013). 23624527 10.1038/ng.2624 PMC3807790

[b31] Ariey F. . A molecular marker of artemisinin-resistant Plasmodium falciparum malaria. Nature 505, 50–55 (2014). 24352242 10.1038/nature12876 PMC5007947

[b32] Duan Y. . Loop Mediated Isothermal Amplification for the Rapid Detection of the F200Y Mutant Genotype of Carbendazim-Resistant Isolates of Sclerotinia sclerotiorum. Plant Dis 100, 976–983 (2016). 10.1094/PDIS-10-15-1185-RE 30686158

[b33] Surabattula R., Vejandla M. P., Mallepaddi P. C., Faulstich K. & Polavarapu R. Simple, rapid, inexpensive platform for the diagnosis of malaria by loop mediated isothermal amplification (LAMP). Exp Parasitol 134, 333–340 (2013). 23562879 10.1016/j.exppara.2013.03.031 PMC7094605

[b34] Nagamine K., Hase T. & Notomi T. Accelerated reaction by loop-mediated isothermal amplification using loop primers. Mol Cell Probes 16, 223–229 (2002). 12144774 10.1006/mcpr.2002.0415

[b35] Tomita N., Mori Y., Kanda H. & Notomi T. Loop-mediated isothermal amplification (LAMP) of gene sequences and simple visual detection of products. Nat Protoc 3, 877–882 (2008). 18451795 10.1038/nprot.2008.57

[b36] Abdul-Ghani R., Al-Mekhlafi A. M. & Karanis P. Loop-mediated isothermal amplification (LAMP) for malarial parasites of humans: would it come to clinical reality as a point-of-care test? Acta Trop 122, 233–240 (2012). 22366670 10.1016/j.actatropica.2012.02.004

[b37] Hanaki K. . Loop-mediated isothermal amplification assays for identification of antiseptic- and methicillin-resistant Staphylococcus aureus. J Microbiol Methods 84, 251–254 (2011). 21167878 10.1016/j.mimet.2010.12.004

[b38] Qi J. . A loop-mediated isothermal amplification method for rapid detection of the multidrug-resistance gene cfr. Gene 504, 140–143 (2012). 22579470 10.1016/j.gene.2012.04.049

[b39] Qi J. . A loop-mediated isothermal amplification method for rapid detection of NDM-1 gene. Microb Drug Resist 18, 359–363 (2012). 22416993 10.1089/mdr.2011.0220

