# Supplementary material for: Establishment and application of a novel isothermal amplification assay for rapid detection of chloroquine resistance (K76T) in Plasmodium falciparum
Source: Sci Rep. 2017 Jan 30;7:41119. doi: 10.1038/srep41119 (PMC5278370; doi:10.1038/srep41119)
Supplement: Supplementary Information [file srep41119-s1.doc]

**Supplementary information**

**Establishment and application of a novel isothermal amplification assay for rapid detection of chloroquine resistance (K76T) in *Plasmodium falciparum***

**Madhvi Chahar, Neelima Mishra, Anup Anvikar, Rajnikant Dixit & Neena Valecha**

**Division of Epidemiology & Clinical Research, National Institute of Malaria Research, Sector-8 Dwarka, New Delhi, India-110077**

**Correspondence and requests for materials should be addressed to N.V. (email:neenavalecha@gmail.com)**

**Table S1. LAMP primers targeting K76T codon of *Pfcrt* gene.** Five set of target specific in-house designed primers of *Pfcrt* gene region indicated as forward inner primer (five set: FIPM1, M2, M3, M4 and M5) and one backward inner primer (BIP) with two outer primers F3, B3: At the initial stage target sequence was recognized by six independent sequences (F1c, F2, F3, B1c, B2 and B3) and the inner primers (FIP, BIP) recognized by four independent sequences (F1c, F2, B1c and B2), target mutation was inserted in the given sets of FIP primers.

| LAMP primer sequence (5’-3’)^a^ | Use |
| --- | --- |
| F3: GATGGCTCACGTTTAGGTG | Forward outer primer |
| B3: TCTTACTTTTGAATTTCCCTT | Backward outer primer |
| BIP:GTA----------------------------------------------------------AATAA* | Backward inner primer |
| *Pfcrt* FIPM1: **GA**ATTCATTACACATACACTTCTTGTCTTGGTAAATGTGCT | Forward inner primer To distinguish the CQ resistant *P. falciparum* having K76T mutation |
| *Pfcrt* FIPM2: **GTT**TTCATTACACATACACTTCTTGTCTTGGTAAATGTGCT |
| *Pfcrt* FIPM3:**GGA**ATTCATTACACATACACTTCTTGTCTTGGTAAATGTGCT |
| *Pfcrt* FIPM4:**G**TA------------------------------------------------------GCT* |
| *Pfcrt* FIPM5:**CG**TATTCATTACACATACACTTATTCTTGTCTTGGTAAATGTGCT |

^a^ Nucleotides in edges are adapted from the *Pfcrt* gene sequence of *P. falciparum*. Nucleotides in bold are mismatches inserted manually to differentiate the CQ resistant *P. falciparum* (mutation at codon K76T) from that of the wild type.

* Patent Pending

**Table S2.** *P. falciparum* samples used in repeatability test of LAMP and their validation by gold standard Sequencing method.

| **Serial No.** | ***P. falciparum* samples** | **Description of genotype** | **Region** | **Resistance profile^a^** | **LAMP^b^** | **Sequencing^c^** |
| --- | --- | --- | --- | --- | --- | --- |
| 1 | TI-01 | Mutation at codon 76 of *Pfcrt* gene (K76T→AAA-ACA) | Tripura, India | CQ^R^ | + | + |
| 2 | TI-02 | Mutation at codon 76 of *Pfcrt* gene (K76T→AAA-ACA) | Tripura, India | CQ^R^ | + | + |
| 3 | TI0-3 | Mutation at codon 76 of *Pfcrt* gene (K76T→AAA-ACA) | Tripura, India | CQ^R^ | + | + |
| 4 | TI-04 | Wild type | Tripura, India | CQ^S^ | - | - |
| 5 | TI-05 | Mutation at codon 76 of *Pfcrt* gene (K76T→AAA-ACA) | Tripura, India | CQ^R^ | + | + |
| 6 | TI-06 | Mutation at codon 76 of *Pfcrt* gene (K76T→AAA-ACA) | Tripura, India | CQ^R^ | + | + |
| 7 | TI-07 | Mutation at codon 76 of *Pfcrt* gene (K76T→AAA-ACA) | Tripura, India | CQ^R^ | + | + |
| 8 | TI-08 | Mutation at codon 76 of *Pfcrt* gene (K76T→AAA-ACA) | Tripura, India | CQ^R^ | + | + |
| 9 | TI-09 | Mutation at codon 76 of *Pfcrt* gene (K76T→AAA-ACA) | Tripura, India | CQ^R^ | + | + |
| 10 | TI-10 | Wild type | Tripura, India | CQ^S^ | - | - |
| 11 | TI-11 | Mutation at codon 76 of *Pfcrt* gene (K76T→AAA-ACA) | Tripura, India | CQ^R^ | + | + |
| 12 | TI-12 | Mutation at codon 76 of *Pfcrt* gene (K76T→AAA-ACA) | Tripura, India | CQ^R^ | + | + |
| 13 | TI-13 | Mutation at codon 76 of *Pfcrt* gene (K76T→AAA-ACA) | Tripura, India | CQ^R^ | + | + |
| 14 | TI-14 | Wild type | Tripura, India | CQ^S^ | - | - |
| 15 | TI-15 | Mutation at codon 76 of *Pfcrt* gene (K76T→AAA-ACA) | Tripura, India | CQ^R^ | + | + |
| 16 | TI-16 | Wild type | Tripura, India | CQ^S^ | - | - |
| 17 | TI-17 | Mutation at codon 76 of *Pfcrt* gene (K76T→AAA-ACA) | Tripura, India | CQ^R^ | + | + |
| 18 | TI-18 | Wild type | Tripura, India | CQ^S^ | - | - |
| 19 | TI-19 | Mutation at codon 76 of *Pfcrt* gene (K76T→AAA-ACA) | Tripura, India | CQ^R^ | + | + |
| 20 | TI-20 | Mutation at codon 76 of *Pfcrt* gene (K76T→AAA-ACA) | Tripura, India | CQ^R^ | + | + |
| 21 | TI-21 | Mutation at codon 76 of *Pfcrt* gene (K76T→AAA-ACA) | Tripura, India | CQ^R^ | + | + |
| 22 | TI-22 | Mutation at codon 76 of *Pfcrt* gene (K76T→AAA-ACA) | Tripura, India | CQ^R^ | + | + |
| 23 | TI-23 | Wild type | Tripura, India | CQ^S^ | - | - |
| 24 | TI-24 | Mutation at codon 76 of *Pfcrt* gene (K76T→AAA-ACA) | Tripura, India | CQ^R^ | + | + |
| 25 | TI-25 | Mutation at codon 76 of *Pfcrt* gene (K76T→AAA-ACA) | Tripura, India | CQ^R^ | + | + |
| 26 | TI-26 | Wild type | Tripura, India | CQ^S^ | - | - |
| 27 | TI-27 | Mutation at codon 76 of *Pfcrt* gene (K76T→AAA-ACA) | Tripura, India | CQ^R^ | + | + |
| 28 | TI-28 | Mutation at codon 76 of *Pfcrt* gene (K76T→AAA-ACA) | Tripura, India | CQ^R^ | + | + |
| 29 | TI-29 | Mutation at codon 76 of *Pfcrt* gene (K76T→AAA-ACA) | Tripura, India | CQ^R^ | + | + |
| 30 | TI-30 | Mutation at codon 76 of *Pfcrt* gene (K76T→AAA-ACA) | Tripura, India | CQ^R^ | + | + |
| 31 | TI-31 | Wild type | Tripura, India | CQ^S^ | - | - |
| 32 | TI-32 | Mutation at codon 76 of *Pfcrt* gene (K76T→AAA-ACA) | Tripura, India | CQ^R^ | + | + |
| 33 | TI-33 | Mutation at codon 76 of *Pfcrt* gene (K76T→AAA-ACA) | Tripura, India | CQ^R^ | + | + |
| 34 | TI-34 | Mutation at codon 76 of *Pfcrt* gene (K76T→AAA-ACA) | Tripura, India | CQ^R^ | + | + |
| 35 | TI-35 | Mutation at codon 76 of *Pfcrt* gene (K76T→AAA-ACA) | Tripura, India | CQ^R^ | + | + |
| 36 | TI-36 | Mutation at codon 76 of *Pfcrt* gene (K76T→AAA-ACA) | Tripura, India | CQ^R^ | + | + |
| 37 | TI-37 | Wild type | Tripura, India | CQ^S^ | - | - |
| 38 | TI-38 | Mutation at codon 76 of *Pfcrt* gene (K76T→AAA-ACA) | Tripura, India | CQ^R^ | + | + |
| 39 | TI-39 | Mutation at codon 76 of *Pfcrt* gene (K76T→AAA-ACA) | Tripura, India | CQ^R^ | + | + |
| 40 | TI-40 | Mutation at codon 76 of *Pfcrt* gene (K76T→AAA-ACA) | Tripura, India | CQ^R^ | + | + |
| 41 | TI-41 | Wild type | Tripura, India | CQ^S^ | - | - |
| 42 | TI-42 | Mutation at codon 76 of *Pfcrt* gene (K76T→AAA-ACA) | Tripura, India | CQ^R^ | + | + |
| 43 | TI-43 | Mutation at codon 76 of *Pfcrt* gene (K76T→AAA-ACA) | Tripura, India | CQ^R^ | + | + |
| 44 | TI-44 | Wild type | Tripura, India | CQ^S^ | - | - |
| 45 | TI-45 | Mutation at codon 76 of *Pfcrt* gene (K76T→AAA-ACA) | Tripura, India | CQ^R^ | + | + |
| 46 | TI-46 | Wild type | Tripura, India | CQ^S^ | - | - |
| 47 | TI-47 | Mutation at codon 76 of *Pfcrt* gene (K76T→AAA-ACA) | Tripura, India | CQ^R^ | + | + |
| 48 | TI-48 | Wild type | Tripura, India | CQ^S^ | - | - |
| 49 | TI-49 | Wild type | Tripura, India | CQ^S^ | - | - |
| 50 | TI-50 | Mutation at codon 76 of *Pfcrt* gene (K76T→AAA-ACA) | Tripura, India | CQ^R^ | + | + |
| A* | 3D7 | Wild type control | Malaria parasite bank | CQ^S^ | - | - |
| B* | NF54 | Wild type control | Malaria parasite bank | CQ^S^ | - | - |
| C* | RKL9 | Mutant Control | Malaria parasite bank | CQ^R^ | + | + |
| D* | MRC2 | Wild type control | Malaria parasite bank | CQ^S^ | - | - |

^a^ CQ^R^  and CQ^S^ Indicate that the *P. falciparum* sample is resistant and sensitive to chloroquine respectively

^b^ and ^c^ ‘+’ an ‘– ’ represent the positive and negative results by LAMP and Sequencing

*Indicates the control samples of *P. falciparum*
